# Genetic and non-genetic parameters of replacement rate component traits in Holstein Friesian cattle

**DOI:** 10.1186/2193-1801-2-581

**Published:** 2013-10-31

**Authors:** Gebeyehu Goshu, Harpal Singh

**Affiliations:** College of Veterinary Medicine and Agriculture, Addis Ababa University, P.O. Box 34, Debre Zeit, Ethiopia

**Keywords:** Heritability-Holstein-replacement rate-fixed factors, Holeta

## Abstract

Records on 3092 pregnancies distributed over a period of 24 years (1986 to 2010) were used to estimate genetic and non genetic parameters of threshold traits in Holstein Friesian. Parity, season and year of calving were included in the model to estimate their effect on replacement traits. A total of 105 sires’ records were used to study the genetic component of the characters. The overall averages for abnormal and normal births, male–female sex ratios, mortality and culling rate in females up to age at first calving and female replacement rates based on female births and total pregnancies were estimated as 12.0% and 88.0%, 52.5% and 47.5%, 23.0% and 7.0% and 70.0% and 29.0% respectively. The effects of parity and year of calving on above traits were found to be significant, except parity effects on culling rate and replacement rate based on total pregnancies, which were non-significant. The season effects for all traits were non-significant. Average 3.45 pregnancies were required to produce one heifer that becomes replacement of the old and low producer cow. The heritability culling and replacement rate from total pregnancy were 0.71 and 0.66 suggesting sufficient additive genetic variance for selecting sires in these traits. Better feeding and health management could reduce mortality and force culling female calves.

## Introduction

Threshold traits are a special class of characters which are qualitative on phenotypic scale but they are, on the contrary, affected by polygenic as well as by environment. Replacement rate in a cow herd is the function of calf production, which is influenced by incidences of abnormal births, sex ratio, post natal mortality and culling of heifers from birth to the age at first calving (Banik and Naskar 2006). The number of replacements heifers is the most important aspect to the advantage of culling inferior females. The genetic gain to a great extent depends on the heritability of the trait and intensity of selection which mainly depends on the number of replacement heifers entering the herd (Rawal and Tomar 1998). Furthermore, information on genetic and non-genetic parameters of replacement rate component traits is helpful in improving the breeding and management practices in a cow herd. In view of the above an investigation on estimation of genetic variability and effect of some non genetic factors viz. parity, season and year of calving on replacement rate component traits in Holstein Friesian herd was under taken.

## Materials and methods

### Description of the study area

Holeta Cattle Genetic Improvement Center is located 33 km west of Addis Ababa, in West Shoa Zone of Oromia Regional state. The center lies at longitude 38º 30′ E and latitude 9º 3′ N and at about 2400 meters above sea level. The site is characterized by cool sub-tropical climate with mean maximum and minimum temperatures of 22.3°C and 6.16°C, respectively with mean relative humidity of 59%. The mean annual rainfall ranged from 818 to 1247 with an average of 1014 mm. The seasons are classified in to dry, short rainy, and long rainy which last from October to February, March to May and June to September, respectively (HARC Holeta Agricultural Research Center 2008).

### Herd management

Cows grazing on native pasture from 8:00 am to 3:00 pm during the rainy season and they are allowed to forage irrigated pasture from February to May. Milking cows were grouped according to their milk production classes as high (>14 liters), medium (8 to 14 liters) and low (< 8 liters) yielding and provided with additional 0.5 kg concentrate per liter of milk, while pregnant dry cows are supplied with 3 kg of concentrates per day in the last two months of pregnancy. The concentrate mix was composed of 30% wheat middling, 30% noug seed cake (*Guizeta abysinica*), 25% wheat bran, 10% corn, 4% limestone and 1% salt. All animals had free access to water.

Calves were separated from their dams after birth and allowed to receive colostrums for the first 5 days of their age. Bucket-feeding of whole milk continued until weaning at 120 days of age. They were given 5, 4, 3, and 2 liters of milk per day from 5 to 65; 66 to 85; 86 to 105; and 106 to 120 days of ages, respectively. Besides, some concentrates and hay starting from the age of 15 days were provided.

Most of the cows were served at first observed heat after calving. Heifers were inseminated after maintaining 280 kg of body weight. Heat detections were routinely followed three times in a day, i.e. early in the morning after milking; in the resting period in mid day; and in the afternoon before milking. Heat detections were carried out by herd attendants and AI technicians. Services were given after the AI technicians confirmed the heat status and then pregnancy diagnosis is conducted by manual rectal palpation after three months of last service.

Regular vaccinations against contagious bovine pleura-pneumonia, lumpy skin disease, anthrax, and blackleg, foot and mouth disease and pasteurellosis were given and treatments provided when incidence of cases observed. Culling was practiced as a result of fertility failures, chronic mastitis, tuberculosis, old ages, and emaciations and in some cases due to low daily milk production.

### Data description and analysis

Abnormal birth includes stillbirth and abortion. A calf born dead between 260 days and full term or calf died within 24 hrs after birth is designated as stillbirth while abortion the loss of the fetus between the age of 42 days and approximately 260 days.

Sex ratio is defined as the probability of a cow at a given lactation producing a live female calf. Mortality is the probability of a female calf died before reaching to the age at first caving. Culling is the probability of a female calf culled before reaching to the age at first caving. Replacement rate is defined as proportion of young females that become replacements to that of the total females born and of total pregnancies in particular year

A total of 3092 calving collected over 24 years from 1986 to 2010 from 105 Friesian sires were used. In this study only singleton births were considered. Twin births were few and removed from the analysis. The traits considered were abnormal and normal births, sex ratios, mortality, and culling and survival rate up to the age at first calving and replacement rates based on female calves born and total pregnancies. The fixed effects studied were parity, season and year of calving. Proportions were estimated for each trait and transformed to arcsine and analyzed using the SAS procedures of General Linear Model (SAS Statistical Agricultural System 2002).


Where,

Y_ijkl_ = i^th^ observation (replacement rate/component traits) from a cow belonging to k^th^ parity, calved in j^th^ season of i^th^ year;

μ = overall mean;

P_i_ = effect of i^th^ parity of lactation (1… 8^+^)

Q_k_ = effect of k^th^ year of calving (1… 24)

S_j_ = effect of j^th^ season of calving (1, 2, 3)

e_ijkl_ = random error specific to the particular observation and assumed to be normally and independently distributed with mean zero and variance σ^2^ ie.σ^2^ ~ NID(0, σ^2^_e_).

Sex ratio is expected to be 1:1. The equality of observed overall sex ratio (percent male birth) with expected overall sex ratio was tested by Chi square (*χ*^2^) test.

Random model was used to study the effect of sires on the different threshold traits. Tomar et al. (1991) devised a method for genetic analysis of proportion data without transformation by conducting the analysis of variance using the following method.


Where,

n_i_ = number of total progenies of i^th^ sire

a_i_ = number of affected progenies of i^th^ sire

N = ∑n_i_

p_i_ = a_i_ / n_i_ = average incidence of the trait among progenies of i^th^ sire

q_i_ = 1- p_i_

Sire component of variance (σ^2^_s_) was estimated from the mean squares of between sire (MS_s_) and within sire component of variance (σ^2^_w_) using the following formula:


Where,


Where,

S = number of sires;

N = total number of observations;

n_i_ = number of observations for i^th^ sire.

Heritability was estimated using paternal half-sib correlation methods using least squares analysis of variance for different traits.


And,


Where,

*σ*^2^*s* = sire component of variance

*σ*^2^*w* = *σ*^2^*e* = error variance

Standard error of heritability estimated according to Tomar et al. (1991).

## Results

### Averages of replacement rate component traits

The overall averages were 12.0% and 88.0%, 52.5% and 47.5%, 23.0% and 7.0% and 70.0% and 29.0% for abnormal births, normal births, sex ratios, mortality, culling, replacement rate based on female births and total pregnancy, respectively (Table 1). Parity and year of calving were found to be significant, except parity effects on culling rate and replacement rate based on total pregnancies, which were non-significant. The season differences for all the traits observed were non-significant.

**Table 1:** **Least squares means for the effect of birth season, parity and year on replacement rate and component traits at Holta Bull Dam Station**

Source	Total preg.	Proportion of birth from total pregnancy		Proportion from normal births (sex ratio)		Proportion from female calves birth		Female replacement rate from	
		Abnormal	Normal	Male Births	Female births	Died	Culled	Female births	Total Preg.
Overall	3092	360(0.12)	2732(0.88)	1435(0.525)	1297(0.475)	303(0.23)	90(0.07)	904(0.70)	0.29
Season		NS	NS	NS	NS	NS	NS	NS	NS
Dry	1368	172(0.13)	1198(0.88)	615(0.51)	583(0.49)	142(0.24)	36(0.06)	405(0.70)	0.30
Short	740	83(0.11)	656(0.89)	355(0.54)	301(0.46)	66(0.22)	20(0.07)	215(0.71)	0.29
Long	984	105(0.11)	878(0.89)	465(0.53)	413(0.47)	95(0.23)	34(0.08)	284(0.69)	0.29
Parity		*	*	***	*	*	NS	***	NS
1	898	141(0.16)	757(0.84)	391(0.52)	366(0.48)	97(0.27)	22(0.06)	247(0.67)	0.28
2	660	70(0.11)	590(0.89)	329(0.56)	261(0.44)	61(0.23)	16(0.06)	184(0.70)	0.28
3	493	43(0.09)	450(0.91)	245(0.54)	205(0.46)	46(0.22)	14(0.07)	145(0.71)	0.29
4	373	34(0.09)	339(0.91)	169(0.50)	170(0.50)	35(0.21)	18(0.11)	117(0.69)	0.31
5	258	32(0.12)	226(0.88)	111(0.49)	115(0.51)	30(0.26)	9(0.08)	76(0.66)	0.29
6	169	17(0.10)	152(0.90)	75(0.49)	77(0.51)	10(0.13)	6(0.08)	61(0.79)	0.36
7	113	13(0.12)	100(0.88)	49(0.49)	51(0.51)	14(0.27)	3(0.06)	34(0.67)	0.30
8+	128	12(0.09)	116(0.91)	66(0.57)	52(0.43)	10(0.19)	2(0.04)	40(0.77)	0.32
Year		***	***	***	***	***	***	***	***
1986-88	70	4(0.06)	66(0.94)	38(0.58)	28(0.42)	0(0.0)	0(0.0	28(1.0)	0.40
1989	71	6(0.08)	65(0.92)	39(0.60)	26(0.40)	0(0.0)	0(0.0)	26(1.00)	0.37
1990	89	11(0.12)	78(0.88)	42(0.54)	36(0.46)	0(0.0)	0(0.0)	36(1.00)	0.40
1991	52	8(0.16)	44(0.84)	21(0.48)	23(0.52)	0(0.0)	0(0.0)	23(1.00)	0.44
1992	39	7(0.19)	32(0.81)	12(0.37)	20(0.63)	0(0.0)	0(0.0)	20(1.00)	0.51
1993	117	10(0.08)	107(0.92)	48(0.45)	59(0.55)	7(0.12)	0(0.0)	51(0.88)	0.44
1994	192	28(0.15)	165(0.85)	84(0.51)	81(0.49)	12(0.15)	3(0.04)	66(0.81)	0.34
1995	123	8(0.07)	115(0.93)	62(0.54)	53(0.46)	13(0.25)	2(0.03)	38(0.72)	0.31
1996	143	19(0.13)	124(0.87)	69(0.56)	55(0.44)	4(0.07)	1(0.02)	50(0.91)	0.35
1997	159	14(0.09)	145(0.91)	81(0.56)	64(0.44)	22(0.34)	2(0.03)	40(0.63)	0.25
1998	177	19(0.11)	158(0.89)	78(0.49)	80(0.51)	17(0.21)	18(0.23)	45(0.56)	0.25
1999	157	9(0.06)	148(0.94)	75(0.51)	73(0.49)	21(0.29)	14(0.19)	38(0.52)	0.24
2000	200	19(0.10)	181(0.90)	100(0.55)	81(0.45)	15(0.19)	10(0.12)	56(0.69)	0.28
2001	190	40(0.21)	151(0.79)	76(0.51)	75(0.49)	17(0.23)	8(0.11)	50(0.67)	0.26
2002	214	25(0.12)	189(0.88)	113(0.60)	76(0.40)	15(0.20)	10(0.13)	51(0.67)	0.24
2003	193	31(0.16)	162(0.84)	83(0.51)	79(0.49)	26(0.33)	5(0.06)	29(0.61)	0.25
2004	175	20(0.11)	155(0.89)	80(0.52)	75(0.48)	20(0.27)	8(0.11)	47(0.63)	0.27
2005	147	17(0.12)	130(0.88)	65(0.50)	65(0.50)	29(0.47)	6(0.09)	30(0.46)	0.20
2006	162	20(0.12)	142(0.88)	74(0.52)	68(0.48)	33(0.49)	1(0.02)	34(0.50)	0.21
2007	175	25(0.14)	150(0.86)	76(0.51)	74(0.49)	36(0.47)	2(0.03)	40(0.49)	0.21
2008	86	4(0.05)	82(0.95)	38(0.46)	44(0.54)	14(0.32)	0(0.0)	30(0.68)	0.35
2009	77	9(0.12)	68(0.88)	41(0.60)	27(0.40)	2(0.07)	0(0.0)	25(0.93)	0.32
2010	84	9(0.11)	75(89.0)	40(0.53)	35(0.47)	0(0.0)	0(0.0)	35(1.00)	0.42

Preg. = pregnancy; * = (P ≤ 0.05); *** = (P ≤ 0.01); NS = not significant.

Figures in parentheses are proportions.

### Heritability of replacement rate component traits

The heritability estimates of replacement rate and its component traits are given in Table 2. Low h^2^ were observed for total calves born, total normal calves born and female calves born which were 0.11 ± 0.018, 0.12 ± 0.018 and 0.10 ± 0.019, respectively. The heritability estimate of selective value i.e. the total female calves reached to milking herd was observed to be 0.66 ± 0.029 and this value was high; however, the value for coefficient of gene replication was negative and very small.

**Table 2:** **Heritability of replacement rate and its component traits in Holstein Friesian at Holeta Bull Dam Station**

Trait	Heritability ± SE
Abnormal births	0.16 ± 0.009
Sex ratio	0.10 ± 0.019
Mortality	0.18 ± 0.029
Culling	0.71 ± 0.029
Replacement rate from female calves born	0.66 ± 0.029

## Discussion

### Averages of replacement rate component traits

The overall average incidence of abnormal birth rates based on total pregnancies was 12.0% while 88.0% of births were normal (Table 1). Similar prenatal death rates were reported by Singh et al. (2002) in Holstein × Sahiwal, Atrey (2003) in Frieswal crosses and Birhanu et al. (2011) in Holstein Friesian. The average abnormal births in Holstein Friesian were higher in dry season than both rainy seasons but the differences were non-significant. This might be because of very low seasonal climatic variations in the area. However, significant season effects on abnormal births in exotic and crossbred cows were observed by Atrey (2003) and Banik and Naskar (2006). The differences in observations could be due to wide differences in seasonal climatic conditions of the locations where the different herds were located.

The parity of calving had significant effects (P ≤ 0.05) on prenatal calf losses due to abnormal birth in Holstein Friesian and agreed with those observed by Kumar et al. (1995), (Mehrotra and Dey 1998b) in exotic and crossbred cattle. The highest incidences of abnormal births (16.0%) in first parity could be related to under developed reproductive system in younger cows by the time of their first calving, resulting in dystocia and prenatal death of calves. This observation was in conformity with Birhanu et al. (2011). The year effects on abnormal births were significant (P ≤ 0.01). This could be attributed to change in management and health care of lactating cows over different years. Similar results were observed by Kumar et al. (1995) and Mukherjee and Tomar (1997b).

The overall male (52.5%) and female (47.5%) sex ratio was significantly deviated from expected sex ratio of 1:1(*χ*^2^_c__(1, 0.01)_ =7.31). Similar findings were reported by Atrey (2003) and Banik and Naskar (2006). Season effects for sex ratio were non-significant indicating that the trait is mostly determined by chance factor and not by environment. Similar observations were also reported by Mukherjee et al. (2000) and Atrey (2003). The male birth varies from 49.0% to 57.0% among different parities and the differences in sex ratios among parities within male and female groups were significant (P ≤ 0.01). These findings were contrary to the reports of Rawal and Tomar (1995) and Mukherjee et al. (2000). The observed higher male calf ratio than the overall average sex ratio in the young and older cows were in agreement with Tomar and Verma (1988a). Such difference in observations for a chance determined trait cannot be explained with valid and logical reasons. The year effects on sex ratios were significant (P ≤ 0.01) and agreed with Kumar et al. (1992) and (Lathwal and Kumar 1993).

The average mortality rate in females from birth to age at first calving was found to be 23.0%. This mortality rate was high and could be related to inadequate health and feeding management of heifers, confirming observations of Birhanu et al. (2011). Higher mortality rates of 35.4 to 63.0% among female calves were reported by Banik and Naskar (2006). The difference in observations of various workers may be attributed to the variations in herd management practices at different farms. Season effects on mortality rates of females from birth to age at first calving were found to be non-significant. Similar non significant season effect on females mortality were observed by (Lathwal and Kumar 1993) and Mukherjee and Tomar (1997b) for various cattle herds. Parity effects on female’s mortality from birth to age at first calving were found to be significant (P ≤ 0.05). The highest mortality was observed in the first parity cows which could be because of lesser skeletal development. Higher mortality of calves from older cows could be attributed to the small sample size. Significant parity effects on calf mortality were reported by Singh et al. (2002) and Atrey (2003). On the contrary, non-significant parity effects on female calf mortality were reported by Mukherjee and Tomar (1997b) and (Tomar and Verma 1988a). Dairy production in tropical countries like Ethiopia is influenced by poor adaptation of exotic breeds and high calf mortality. After studying the dynamics of Friesian herds in four dairy farms Menjo et al. (2009) concluded that 25% of the Holstein Friesian cattle born on the Kenyan large scale farms were lost before reaching to a productive age, indicating the limitations of such animals’ adaptability to the prevailing environmental conditions. Moreover, Moran (2011) reviewed 17 studies documented on mortality of calves in Asia, tropical Africa and south America and summarized that pre-weaning calf mortality ranged from 15 to 25% and often as high as 50%. The main reasons attributed to the death of exotic breeds in these countries were poor adaptation coupled with poor health, feeding and management problems.

The year differences in mortality rates of the Holstein Friesian heifers were found to be highly significant (P ≤ 0.01). This could be attributed to the management and environmental differences over different years and agreed with reports of Rawal and Tomar (1994b), Mukherjee and Tomar (1997b). However, values for the year 2010 should be interpreted cautiously since animals may not get sufficient time for expression of component traits.

The overall female culling rate up to age at first calving, computed from total female calves born was observed to be 7% (Table 1). Lower culling rate in female calves were reported by (Chaudhery et al. 1984) and Singh et al. (1987) for Friesian × indigenous crosses breeds. However, Shahi and Kumar (2006) reported a higher culling rate of 17.8% for Holstein crosses. The observed variations among the reports may be because of the differences in the management practices, standards of culling and replacement need at different livestock farms. Season differences in culling rates of female calves were found to be statistically non-significant. These results agreed with the reports of (Lathwal and Kumar 1993) and Mukherjee and Tomar (1997b). The differences in heifer’s culling rates according to dam’s parity order were non significant and agreed with Singh (2001) and Atrey (2003).

Year differences for culling rates in female calves were highly significant (P ≤ 0.01). This could be resulted from the variations in disease incidences, climatic conditions, management practices, replacement need and availability of replacement heifers over different years. Significant differences over years for culling rate have been reported by (Tomar and Rawal 1996) and Mukherjee and Tomar (1997b).

The overall replacement rate in Holstein Friesian computed from total female calves born was 70.0%. These observations agreed with 68.5% (Tomar and Verma 1988a) and 69.5% (Tomar and Rawal, 1994). However, female replacement rate at Holeta was lower than 92.2% (Tomar and Verma 1988b) and 74.7% Atrey (2003) reported for different cattle herds. The differences due to season of calving in replacement rate from female calf born were found to be non significant. However, significant effects of season of calving on replacement rate on female calf’s basis were reported by Tomar and Verma (1988a) and Atrey (2003). Dams’ parity order was significant and ranged from 66% in 5^th^ parity to 83% in 8^th^ parity. Similar findings were reported by (Lathwal and Kumar 1993) and Tomar and Rawal (1994). Lower replacement rate in some parity could be related to the combined effect of high mortality, culling and high male births.

The year differences (Table 1) in replacement rates basis on female calves born were highly significant (P ≤ 0.01). This could be attributed to the differences in culling and mortality of female calves over different years and supported by the findings of (Lathwal and Kumar 1993) and Atrey (2003). However, Tomar and Verma (1988b) reported non-significant year effects on replacement rate computed from female calves born.

The overall replacement rate computed from total pregnancies was estimated as 29.0%. These observations indicated that about 3 to 4 pregnancies were required to produce one heifer replacement. The present overall replacement rate from total pregnancies was lower than the reports of Tomar and Rawal (1994), and (Tomar and Verma 1988a). The season differences in replacement rates based on total pregnancies was not significant and agreed with Tomar and Rawal (1994). Whereas, Tomar and Verma (1988a) and Atrey (2003) has reported significant season effects on replacement rate based on total pregnancies. The average replacement rate based on total pregnancies among dam’s parities ranged from lowest of 28% in 1^st^ and 2^nd^ parity to 36% in 6^th^ parity. However, no definite trend was observed for replacement rate among dam’s parities and the observed differences were non-significant.

The year effects on replacement rate based on total pregnancies were highly significant (P ≤ 0.01). Significantly lower replacement rates in some years may be because of very high abnormal birth, mortality and culling rates during these years. These findings agreed with Tomar and Rawal (1994) and Atrey (2003).

### Heritability of replacement rate component traits

The low heritability (0.16 ± 0.01) of abnormal birth in Holstein Friesian indicated very small chances of reducing the abnormal birth through selection. This finding was in complete conformity with the reports of (Rawal and Tomar 1996c). However, Rawal and Tomar (1996a) and Atrey (2003) have reported heritability values ranging from 0.042 to 0.10. The low heritability (0.10 ± 0.02) for sex ratio could be due to chance factor and was in agreement to that of 0.095 (Rawal and Tomar 1995). Very low heritability estimates (0.016) for sex ratio have been observed by Lathwal and Kumar (1993) in zebu and 0.017 by Powell et al. (1975) in exotic cattle. These reports showed that the variation in sex ratio was mainly due to non genetic factors consisting of random union of gametes in determination of sex of the calf.

The heritability estimate of mortality in Holstein Friesian female calves was 0.18 ± 0.03 suggesting that selection could be used for reducing the mortality rate in female calves genetically. Very close findings were reported by Atrey (2003). Medium heritability estimate (0.38) was found Lathwal and Kumar (1993) for the mortality in female calves. Improvement in non-genetic factors viz. feeding, health and overall management of heifers could be more helpful in reducing the female calf mortality. The heritability of culling rate in female calves from birth to age at first calving was high (0.71 ± 0.03), suggesting that culling rate in female calves could be altered through selection among the female progenies of the different sires. Moreover, culling in female calves as a result of poor growth, reproductive problems and susceptibility to diseases and parasites might have been influenced by inherited genes related to the genetic makeup of the calf, hence contributing to the high heritability of the trait. However, lower heritability values of culling rate than the present estimate were reported by (Schwenger et al. 1989) and Atrey (2003) for different breeds of cattle. Replacement rate from female calf births had high heritability (0.66 ± 0.03) indicating and sires of the daughter greatly affected the status of female calves reaching to the milking herd. Lower heritability of 0.23 (Mukherjee and Tomar 1997b) has been reported for a tropical breed of cattle. In the present study ANOVA method without transformation was used. Since the method can present negative estimates especially when the error term is high, it is plausible to consider linear threshold model for genetic analysis for big data set.

## Conclusion

From the foregoing discussion, it could be concluded that on an average 3.45 pregnancies are required to produce one heifer that become replacement of the old and low producer cows. Further, sufficient additive genetic variance was available to affect the selection for low incidence of abnormal births, low female calf mortality rate, and low female culling rate.

## Authors’ information

GG: MSc in Animal Production, Assistant Professor, PhD scholar.

HS: PhD, Professor in Animal Breeding, advisor.

## References

[CR1] Atrey RK (2003). Genetics of replacement rate in Frieswal cattle. Ph.D. Thesis.

[CR2] Banik S, Naskar S (2006). Effect of non-genetic factors on replacement rate and its components in Sahiwal cattle. Indian J Anim Sci.

[CR3] Birhanu Y, Fikre L, Gebeyehu G (2011). Calf survival and reproductive performance of Holstein-Friesian cows in central Ethiopia. Trop Anim Hlth Prod.

[CR4] Chaudhery G, Banerjee GC, Guha H (1984). Genetic and environmental causes of variations in the mortality rates among the crossbred calves of Jersey × Hariana and Holstein × Hariana types. Indian J Anim Hlth.

[CR5] HARC (Holeta Agricultural Research Center) (2008). Holeta Agricultural Research center 2008 Annual Report.

[CR6] Kumar A, Lavania GS, Lathwal SS (1992). Sex ratio in crossbred cattle. Indian J Anim Res.

[CR7] Kumar A, Chaudhary SR, Sachdeva GK (1995). Abnormal termination of pregnancies in crossbred cattle. Indian J Dairy Sci.

[CR8] Lathwal SS, Kumar A (1993). Genetics of replacement rate and its components in Red Sindhi cows. Indian J Vet.

[CR9] Mehrotra S, Dey A (1998). Calving pattern and sex ratio in exotic and crossbred cattle reared under suitable climate. Indian J Vet.

[CR10] Menjo DK, Bebe BO, Okeyo AM, Ojango JMK (2009). Survival of Holstein-Friesian heifers on commercial dairy farms in Kenya. Appl Anim Husb Rural Dev.

[CR11] Moran JB (2011). Factors affecting high mortality rate of dairy replacement calves and heifers in the tropics and strategies for their reduction. Asian-Australian J Anim Sci.

[CR12] Mukherjee K, Tomar SS (1997). Genetics of female calf losses up to maturity and replacement rate in crossbred cattle. Indian J Dairy Sci.

[CR13] Mukherjee K, Tomar SS, Singh RB (2000). Variability in sex ratio. J Anim Res.

[CR14] Powell RL, Norman HD, Dickinson FN (1975). Sire differences in sex ratio of progeny. J Dairy Sci.

[CR15] Rawal SC, Tomar SS (1994). Inherited variation in mortality and culling rates in Sahiwal female calves up to maturity. Indian J Anim Sci.

[CR16] Rawal SC, Tomar SS (1995). Genetic study of sex ratio in a herd of Sahiwal cattle. Indian J Dairy Sci.

[CR17] Rawal SC, Tomar SS (1996). Incidence and inheritance of abnormal calving in a herd of Sahiwal cattle. Indian J Vet.

[CR18] Rawal SC, Tomar SS (1996). Incidence and inheritance of type and sex of calf born in Tharparkar cattle. Indian J Dairy Sci.

[CR19] Rawal SC, Tomar SS (1998). Population analysis for loss of cows and replacement index in Tharparkar cattle. Indian J Anim Sci.

[CR20] SAS (Statistical Agricultural System) (2002). Version 9.0.

[CR21] Schwenger B, Mayer M, Simon D (1989). Antagonism between dairy performance and fitness in cattle. Anim Breed Abst.

[CR22] Shahi BN, Kumar D (2006). Factors affecting replacement rate and its components in Jersey-Sahiwal cattle. Indian J Anim Sci.

[CR23] Singh L (2001). Genetics of replacement rate in Karan Fries cattle. Ph.D. Thesis.

[CR24] Singh RN, Mishra RR, Dutta OP, Prabhakarna VT, Malhtora JC (1987). An investigation on the viability among indigenous and crossbred cows with special reference to mortality and culling. Indian J Anim Sci.

[CR25] Singh CV, Kumar D, Singh H (2002). Genetic and non-genetic variation in components of replacement rates in crossbred and Sahiwal cattle. Indian J Diary Sci.

[CR26] Tomar SS, Rawal SC (1994). Replacement rate in Sahiwal herd. Indian J Vet.

[CR27] Tomar SS, Verma GS (1988). Genetic and non genetic variations in components of replacement rates in Tharparkar cattle. Indian J Dairy Sci.

[CR28] Tomar SS, Verma GS (1988). Genetic variability in components of replacement in Karan Fries cattle. Indian J Anim Sci.

[CR29] Tomar SS, Rawal SC (1996). Incidence and inheritance of mortality and culling rates in Tharparkar female calves up to maturity. Indian J Dairy Sci.

[CR30] Tomar SS, Kumar A, Ajay K (1991). New approach for genetic analysis of proportion data without transformation. Asian J Dairy Res.

